# A Planar Ultrawideband Patch Antenna Array for Microwave Breast Tumor Detection

**DOI:** 10.3390/ma13214918

**Published:** 2020-11-02

**Authors:** Amran Hossain, Mohammad Tariqul Islam, Md. Tarikul Islam, Muhammad E. H. Chowdhury, Hatem Rmili, Md. Samsuzzaman

**Affiliations:** 1 Department of Electrical Electronic and Systems Engineering, Universiti Kebangsaan Malaysia, Bangi 43600, Selangor, Malaysia; p102123@siswa.ukm.edu.my (A.H.); p94299@siswa.ukm.edu.my (M.T.I.); 2 Department of Electrical Engineering, Qatar University, Doha 2713, Qatar; 3 Center of Research Excellence in Renewable Energy and Electrical Power Systems (CREPS), King Abdulaziz University, P.O. Box 80204, Jeddah 21589, Saudi Arabia; 4 Patuakhali Science and Technology University, Patuakhali 8602, Bangladesh; samsuzzaman@ukm.edu.my

**Keywords:** ultrawideband, semicircular-shaped, breast tumor, patch antenna, microwave imaging

## Abstract

In this paper, a compact planar ultrawideband (UWB) antenna and an antenna array setup for microwave breast imaging are presented. The proposed antenna is constructed with a slotted semicircular-shaped patch and partial trapezoidal ground. It is compact in dimension: 0.30λ × 0.31λ × 0.011λ, where λ is the wavelength of the lowest operating frequency. For design purposes, several parameters are assumed and optimized to achieve better performance. The prototype is applied in the breast imaging scheme over the UWB frequency range 3.10–10.60 GHz. However, the antenna achieves an operating bandwidth of 8.70 GHz (2.30–11.00 GHz) for the reflection coefficient under–10 dB with decent impedance matching, 5.80 dBi of maximum gain with steady radiation pattern. The antenna provides a fidelity factor (FF) of 82% and 81% for face-to-face and side-by-side setups, respectively, which specifies the directionality and minor variation of the received pulses. The antenna is fabricated and measured to evaluate the antenna characteristics. A 16-antenna array-based configuration is considered to measure the backscattering signal of the breast phantom where one antenna acts as transmitter, and 15 of them receive the scattered signals. The data is taken in both the configuration of the phantom with and without the tumor inside. Later, the Iteratively Corrected Delay and Sum (IC–DAS) image reconstructed algorithm was used to identify the tumor in the breast phantom. Finally, the reconstructed images from the analysis and processing of the backscattering signal by the algorithm are illustrated to verify the imaging performance.

## 1. Introduction

Globally, breast cancer is stated to be a foremost reason for women’s death. Each year, approximately 25% of new cases of breast cancer are identified [1]. Breast cancer begins by owing to the existence of malignant cells in the breast tissues. Currently, this disease is considered a major women’s health problem worldwide [2], and a reliable early stage diagnosis is seen as the key aspect in treating it. If it is possible to detect breast cancer in early stages by using a reliable technique, then the treatment may attain a survival percentage of up to 97% [3]. The standard technologies in medical imaging such as Computed Tomography (CT), Magnetic Resonance Imaging (MRI), Ultrasound, and X-ray mammography are usually used for detecting cancerous breast cells or tumors [4,5]. The main advantages of CT scanning are (1) particularly suitable for identifying acute and chronic changes to the internal structure of the human body, (2) rapid acquisition of images, (3) excellence in diagnosing diseases of the large vessels [6]. However, the main disadvantages of CT scanning are (1) exposure to radiation, and (2) performance deprivation [6,7]. The prime benefits of MRI are (1) excellent in diagnosis of disease of the large vessels, (2) more accurate information about scanning object, and (3) MRI helps doctors to evaluate different parts of the human body, as well as to detect the presence of a particular disease (e.g., breast tumor) [8]. However, the disadvantages of this technology are that (1) it takes a long time for testing, (2) the cost is higher, and (3) the compression of body parts is uncomfortable, and ionizing radiation poses a high risk [8,9]. The ultrasound technique is better for a particular domain, and it has no ionizing radiation, but the main disadvantages are that (1) it produces low-resolution images, (2) image perfection depends on the technicians, and (3) it is hard to identify the deep-lying tumor [10]. The X-ray mammography technique has some advantages such as (1) it is a simple technique and cheaper in terms of cost, (2) it produces lower radiation compared to a CT scan, and (3) it helps to detect alien objects inside the bone. However, the main limitations are (1) it increases high false-negative rates, (2) low-quality image with low sensitivity, as well as (3) due to short ionizing radiation, it increases the cancer risk [11]. Finally, a well-known limitation of the X-ray is its associated pain. It requires compression of the breast, and many women choose to not go in for screening for this reason. Thus, at present, microwave imaging (MWI) is a promising technique for breast tumor detection due to non-ionizing radiation upshot, efficient, low profile, low cost, and comfort in respect to traditional medical identification technologies such as CT scan, MRI or X-ray. Due to the features of MWI, the researchers are motivated to establish an MWI framework to identify the breast tumor. The fundamental use of MWI is to distinguish the dielectric properties of tumorous and normal cells in the breast tissues. With respect to breast tumors, there is a momentous difference between the dielectric properties of normal breast tissue and a malignant cell (i.e., tumor cell). The malignant breast tumor dielectric properties are higher than that of the normal breast tissue, which is a significant aspect for using the microwave as breast imaging for the detection of malignant cells [12].

However, microwave antennas played a prime role in this technology and are able to easily distinguish the small pulse alterations from the variations of the electrical properties of human tissues. In this imaging technique, the antenna transmits microwave pulses across the target object. On the other hand, the receiver receives the scattered pulses in several directions. Owing to having dissimilar dielectric characteristics of the different tissue, the received scattered pulse delivers significant data about pulse propagation across the cells. Then, applying different algorithms on received scattered signals, it is possible for the image of the specific object, such as a breast tumor, to be plotted. However, the main objective of MWI is to design a trustworthy, low-cost, low-profile imaging system that can be used for medical diagnosis purposes. At present, in MWI technology, the ultrawideband (UWB) antenna is used to detect a tumor in the breast because of its extraordinary features such as high data rate, high resolution, low complexity, low profile, lightweight, good efficiency, and radiation directivity [13]. The wideband feature provides high-quality resolution images and precise localization of a target object for imaging [14]. For attaining improved outcomes in the breast imaging platform, UWB antennas are a suitable candidate [15,16]. Therefore, the design of the appropriate UWB antenna is a requirement in the breast MWI system. However, a number of ultrawideband antennas have been designed for ultrawideband applications, such as the planar inverted cone antenna [17], combinations of the monopole and slot antenna [18], and the volcano smoke antenna [19] for ultrawideband applications. In addition, several UWB antennas are proposed for MWI; for instance, parasitic resonator-based UWB antennas [20,21,22], slotted UWB antennas [23], tunable UWB antennas [24], sensor-based UWB antennas [13], different types of Vivaldi antennas [4,25,26,27,28,29,30,31], directional UWB antennas [32,33,34,35], CPW UWB antennas [36], EBG-based UWB antennas [37,38], polarized-UWB antenna arrays [39,40], and different types of patch antennas [13,41]. However, a parasitic resonator-based UWB antenna was proposed for breast imaging in [21]. In this approach, antenna size is 30 × 29 × 1.6 mm^3^, but its gain is comparatively low, with low imaging resolution. Different kinds of Vivaldi antennas were designed in [4,25,26,27,28,29,30,31] with comparatively high resolution, but the size is large concerning the proposed antenna. A CPW-fed UWB antenna was proposed in [30] for breast imaging, with two rotating antenna array platforms, but the gain is poor, the scanning image resolution is not high, and the antenna size is very large. In [37,38], EBG-based UWB antennas were proposed for ultrawideband applications. Due to the inefficient design of the antenna, the gain was relatively poor, and scanning image resolution was not satisfactory.

In this paper, a compact planar UWB antenna for breast imaging is presented. Significantly, a UWB antenna should have the ability to produce more than one resonance in MWI applications [20]. This feature is vital for producing a high-quality image and good penetration in the depth portion of the object [27]. Besides, the antenna should also have a higher gain, wider bandwidth, high efficiency, and directional radiation pattern than microstrip patch antennas. Hence, in this article, a new planar ultrawideband antenna has been introduced and clarified its overall characteristics. The dimension of the antenna is 0.30λ × 0.31λ × 0.011λ, where λ is the wavelength of the lowest operating frequency. It attains an operational bandwidth of 2.30 GHz to 11.00 GHz, with a maximum gain of 5.80 dBi and directional radiation. The antenna produces multiple resonance frequencies, directional radiation, and higher fidelity factor (>80%), which are prerequisites for microwave breast imaging. The time-domain and frequency-domain features of the antenna have been investigated to evaluate antenna performance. Sixteen antenna arrays are used and placed vertically and horizontally, surrounding the imaging arrangement for the breast phantom imaging to detect the tumor inside the breast. Finally, the simulation environment and the imaging outcomes are demonstrated in the results section.

## 2. Design Methodology of the Proposed Antenna

**Figure 1:**
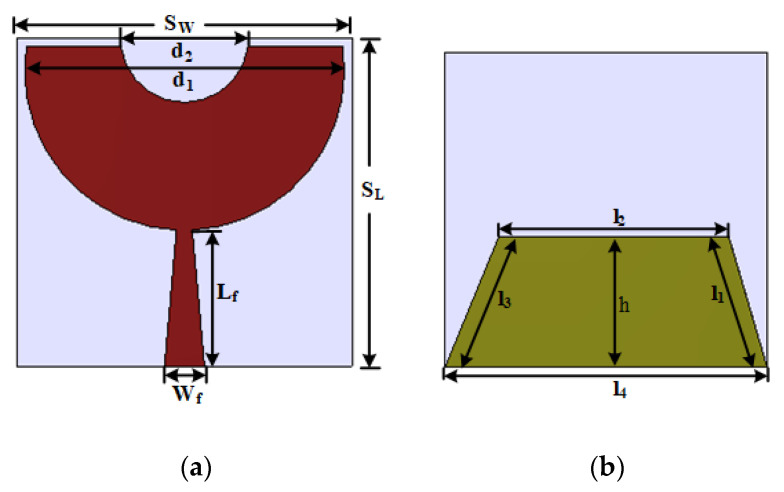
Geometric structure of the proposed antenna: (**a**) Front view; (**b**) Rear view.

**Figure 2:**
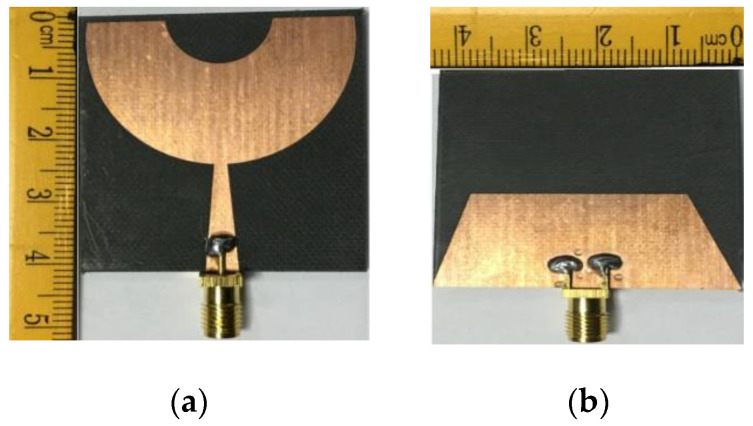
Fabricated prototype: (**a**) Front view; (**b**) Rear view.

Significantly, a UWB antenna should have the ability to produce more than one resonance in MWI applications [20]. This feature is vital for producing high-quality images and good penetration in the depth portion of the object [27]. The antenna should also have higher gain, wider bandwidth, high efficiency, and a directional radiation pattern. Hence, in this article, a new planar ultrawideband antenna has been introduced and its overall characteristics are clarified. The geometry and fabricated prototype of the antenna are depicted in Figure 1 and Figure 2, respectively. The antenna is designed on the Rogers RT5880 dielectric substrate material. The thickness (*T_h_*), relative permittivity ($\varepsilon_{r}$), and loss tangent (δ) of the substrate is 1.575 mm, 2.2 and 0.0009, respectively. The magenta and green colors in the graphical design are considered copper with 0.035 thickness. The proposed antenna has a slotted semicircle-shaped radiating patch along with a trapezoidal-shaped ground plane, which is coupled to a 50 Ω tapered-shaped microstrip-fed line. The tapered-shaped feed line is designed for impedance matching, and a small semicircle-shaped slot is cut out from the radiating patch to enhance the radiation directivity. A partial trapezoidal ground plane is attached to the ground for increasing the entire bandwidth. All design parameters of the prototype are shown in Table 1.

## 3. Parametric Study of the Proposed Antenna

**Figure 3:**
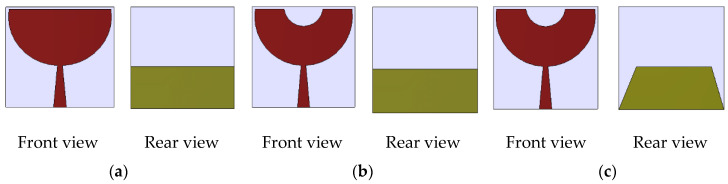
Different modifications of the proposed prototype: (**a**) Normal patch design; (**b**) Slotted patch design; (**c**) Proposed design.

**Figure 4:**
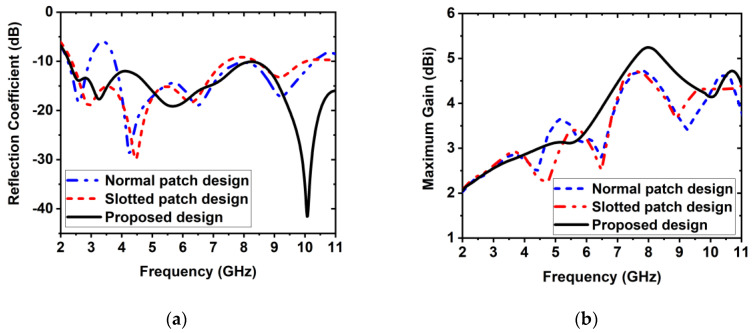
The effect of different design structure on: (**a**) S_11_(reflection coefficient); (**b**) Maximum gain.

**Figure 5:**
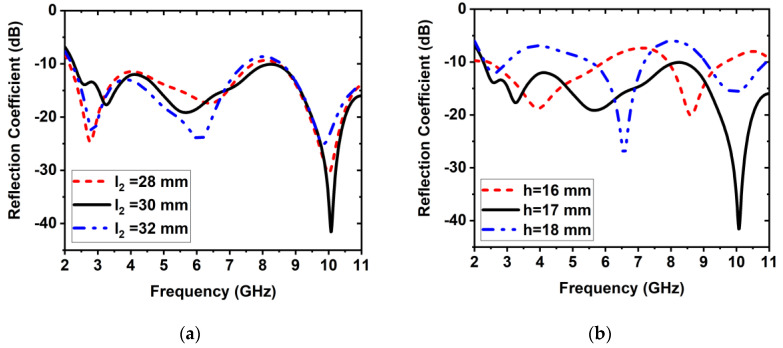
The effect of the reflection coefficient: (**a**) When length l_2_ = 28 mm, 30 mm, and 32 mm; (**b**) When height h = 16 mm, 17 mm, and 18 mm.

There are 11 parameters in the antenna design; those are used, and their values are optimized for attaining the overall operating frequency band. In this work, the preliminary design has been investigated in both HFSS and CST simulators to assess performance of the antenna. The two simulators work on two different methods. Therefore, before fabricating the prototype, we checked the results from both simulators for accuracy of the designed antenna. This matching between both the simulators guarantees that the fabricated prototype will show expected performance while measuring. The various modified structures of the antenna are depicted in Figure 3. The length and width of the antenna are represented by *S_L_* and *S_w_*, respectively. The value of the *S_L_* and *S_w_* is considered 41 mm and 42 mm, which is the optimized size for achieving the operating band from 2.26 GHz to 11.00 GHz. A partial trapezoidal ground is attached to the backplane of the substrate material. The length of the trapezoidal arms of the ground plane is represented by *l_1_*, *l_2_*, *l_3_*, and *l_4_*, and the height of the ground denoted by *h*. The considered values are 17.72 mm, 30 mm, 18.38 mm, and 42 mm, and height is 17 mm, respectively, which is the optimized size for achieving the operating band from 2.26 GHz–11.00 GHz. The dimension of the main semicircle-shaped radiator is represented by *d_1_*. A 15 mm semicircle-shaped slot has been cut off from the central location of the patch to increase the radiation directivity. Figure 4a depicts the reflection coefficient of different modification structures of the prototype. First investigated is that, in normal patch design, the antenna produces four resonances in the frequency ranges from 2.26 GHz to 10.00 GHz, but the reflection coefficient curve goes to upward from −10 dB in between the frequency ranges from 3.00 GHz to 4.00 GHz. So, the operating frequency range is from 2.26 GHz to 3.00 GHz, which does not cover ultrawideband. Second, in the slotted patch design, the antenna produces three resonances at 3.00 GHz, 4.50 GHz, and 6.50 GHz under −15 dB in the frequency ranges of 2.30 GHz to 7.00 GHz. The highest resonance pick has produced under −30 dB at 4.50 GHz. However, the operating frequency range is from 2.30 GHz to 7.00 GHz, and it does not cover an ultrawideband frequency range. Finally, in the proposed design, the antenna produces three resonances at 3.50 GHz, 5.50 GHz, and 10.00 GHz under −18 dB in the frequency range from 2.26 to 11.00 GHz. The highest resonance pick has generated under −40 dB at 10.00 GHz. Therefore, in the proposed design, the antenna’s operating band is from 2.26 GHz to 11.00 GHz, which covers the ultrawideband frequency. The simulated maximum gain of different modification structures of the antenna is depicted in Figure 4b. It is seen that the simulated maximum gain of different structures is 2.50 dBi, 4.50 dBi, and 5.48 dBi, respectively. The overall assessment of the different structure designs is presented in Table 2. The significant effect has been observed on the antenna characteristics such as S_11_ due to the variations of the length *l_2_* and height *h* of the trapezoidal ground plane. Further, the effect of the reflection coefficient is depicted in Figure 5, when *l_2_* and *h* are varied, while other parameters remain constant. It is examined that, when length *l_2_* is deceased 2 mm (i.e., *l_2_* = 28 mm) and other parameters remain constant, then the lower frequency has shifted to 2.23 GHz from 2.26 GHz, and the upper frequency has shifted to 8.00 GHz from 11.00 GHz, operating frequency band is decreased to 8.00 GHz and generated one resonance frequency at 3.00 GHz near to–25 dB. Furthermore, when length *l_2_* is increased 2 mm (i.e., *l_2_* = 32 mm) and other parameters remain constant, the lower frequency shifts to 2.24 GHz from 2.26 GHz, and the upper frequency shifts to 7.00 GHz from 11.00 GHz with two resonances close to −25 dB. However, in these two scenarios, the antenna does not cover ultrawideband range. Therefore, it is noticeable that the proposed length *l_2_* = 30 mm is optimized to get ultrawideband frequency. On the other hand, when ground plane height *h* is decreased 1 mm (i.e., *h* = 16 mm) and other parameters remain constant, then the antenna’s operating frequency range is from 2.26 GHz to 6.00 GHz with single resonance at 4.00 GHz under −15 dB, which does not cover ultrawideband. In this scenario, the upper frequency has shifted to 6.00 GHz from 11.00 GHz. In addition, when height *h* is increased 1 mm (i.e., *h* = 18 mm) and other parameters remain constant, then the antenna generated multiple bands with lower reflection. In this case, the operating frequency range is from 2.26 GHz to 3.00 GHz with single resonance at 2.50 GHz close to −15 dB, which does not cover ultrawideband. In this scenario, the upper frequency has shifted to 3.00 GHz from 11.00 GHz. So, it is concluded that the proposed ground plane height *h* = 17 mm is the optimized value to achieve the operating frequency band from 2.26 GHz to 11.00 GHz.

## 4. Result and Discussion

**Figure 6:**
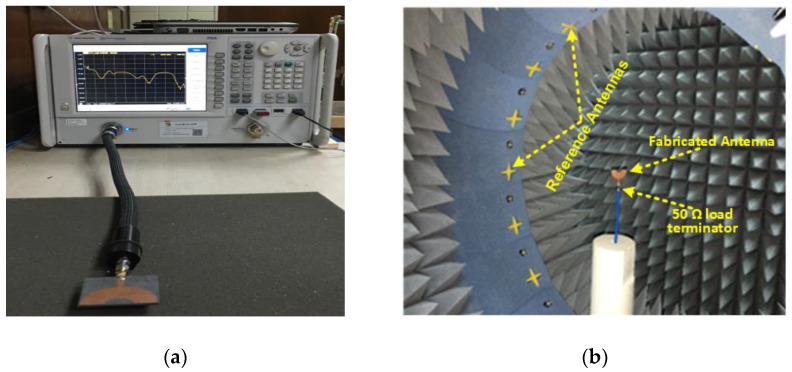
Measurement setup: (**a**) PNA measurement scenario; (**b**) StarLab measurement scenario.

In this section, we discuss the obtained performance results of the antenna during measurement and simulation. The frequency-domain performance and time-domain performance are key factors in evaluating the antenna’s performance. These performances have been examined and simulated through the CST and HFSS simulator and measured by using different types of equipment. However, the measured results of the fabricated prototype are compared with the simulation results to evaluate the performance. The Agilent N5227 PNA network analyzer is used to measure the S_11_(reflection coefficient). It operates within 10 MHz to 67 GHz. The UKM StarLab (Microwave Vision Group, Paris, French) near field antenna measurement system is used to measure the efficiency, gain and radiation pattern of the prototype. After that, primary data is collected in near-field and further converted to far-field by using the SatEnv software (Microwave Vision Group, Paris, France). The measurement setup of the PNA network analyzer (Agilent technologies, Inc., Santa Clara, CA, USA; N5227A 10 MHz–67 GHz) is demonstrated in Figure 6a, and the StarLab measurement setup is shown in Figure 6b.

### 4.1. Performance Analysis in the Frequency Domain 

**Figure 7:**
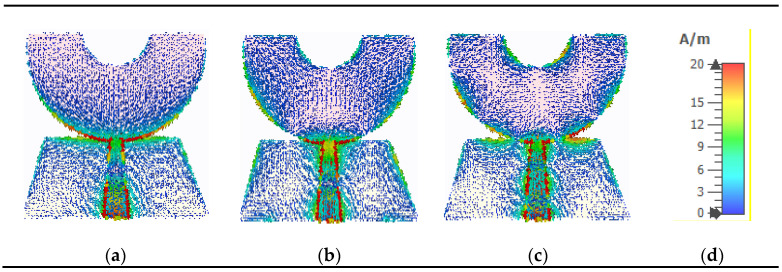
The surface current distribution at: (**a**) 3.50 GHz; (**b**) 5.50 GHz; (**c**) 10.00 GHz and (**d**) scale

**Figure 8:**
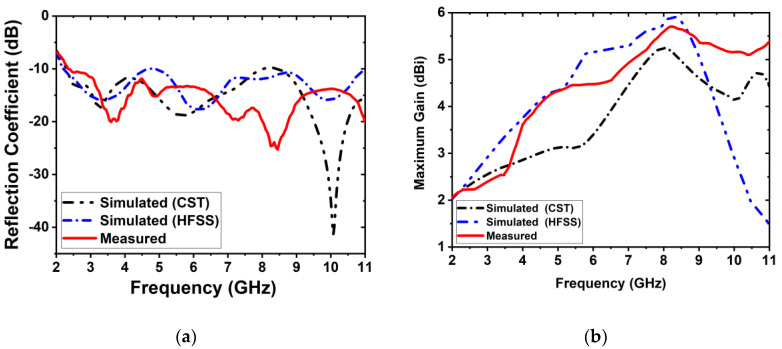
The measured and simulated: (**a**) Reflection coefficient (S_11_) and (**b**) Maximum gain of the prototype.

**Figure 9:**
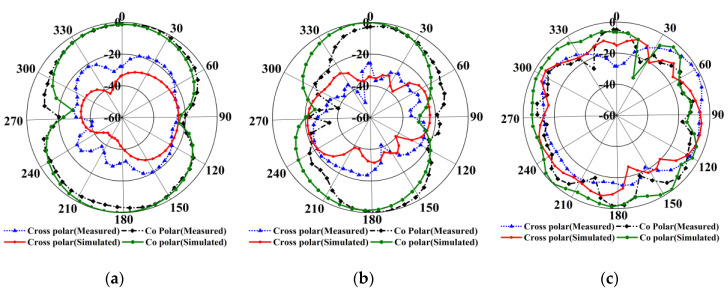
Simulated and measured 2D radiation pattern of E plane (φ = 0) of the proposed antenna at: (**a**) 3.50 GHz; (**b**) 5.50 GHz; (**c**) 10.00 GHz.

To evaluate the antenna performance in the frequency domain, the simulated surface current distribution, simulated and measured reflection coefficient, gain and radiation pattern characteristics are illustrated in this section. The circulation of the surface current of the prototype for three different resonances of 3.50 GHz, 5.50 GHz, and 10.00 GHz is displayed in Figure 7. It is determined that the highest predominant surface current zone of the prototype is the lowermost edge of the semicircle radiating patch, around the feeding line, as well as the top and side edges of the trapezoidal ground plane. It is also seen that at 5.50 GHz and 10.00 GHz frequencies, the moderate current conduction area is in the top semicircle-shaped slot of the patch. Moreover, there exists a slight number of nulls on the radiating patch at higher frequency (i.e., at 10.00 GHz) due to the higher-order current mode. Figure 8a represents the measured and simulated S_11_(reflection coefficient) of the proposed prototype. The simulated results were obtained from both CST and HFSS. The proposed structure attained a −10 dB reflection coefficient of 8.74 GHz (2.26–11.00 GHz) impedance bandwidth. The lower-frequency bandwidth is sensitively affected due to use of the semicircle-shaped slot in the radiating patch and trapezoidal ground plane. However, the measured S_11_ (reflection coefficient) has slightly shifted toward from 2.26 GHz to 2.30 GHz (i.e., 40 MHz) concerning the simulated result. So, the measured operating frequency range is from 2.30 GHz to 11.00 GHz (i.e., Bandwidth is 8.70 GHz). From the measurement outcomes, it is observed that resonance frequencies have produced at 3.60 GHz, 4.90 GHz, and 8.50 GHz. The slight mismatch between the simulated and measured results may be due to fabrication and soldering tolerance. Otherwise, the simulated and measured outcomes exhibited good agreements through the entire bandwidth. Figure 8b presents the measured and simulated gain curve. The measured maximum gain is achieved as 5.80 dBi at 8.26 GHz. However, the measured and simulated 2D radiation patterns, together with co-polar and cross-polar of E-plane when phi(φ) = 0, is shown in Figure 9 for three resonances of 3.50 GHz, 5.50 GHz, and 10.00 GHz. It is determined that the simulation and measured outcomes show good agreement. Moreover, the proposed prototype illustrates stable directional radiation. It is also determined that at higher frequencies, antenna radiation shows the omnidirectional characteristics with some side lobes because at upper frequencies, many nulls may increase in current circulation that produces few side lobes.

### 4.2. Performance Analysis in the Time Domain

It is noticeable that frequency-domain analysis alone may not ensure the antenna’s performance. Therefore, for the assessment of the accurateness of the proposed antenna in the MWI system, it is essential to investigate the time-domain performance, including the input-output pulse waveform and fidelity factor. To investigate input-output pulse characteristics, two types of setups, such as face-to-face and side-by-side, are considered to evaluate the time-domain performance of the proposed prototype. In both cases, the distance of the two antennas is assumed to be 250 mm. Overall, it is determined that, in both setups, the waveforms are nearly identical for transmitted and received signals, although it spread slightly.

**Figure 10:**
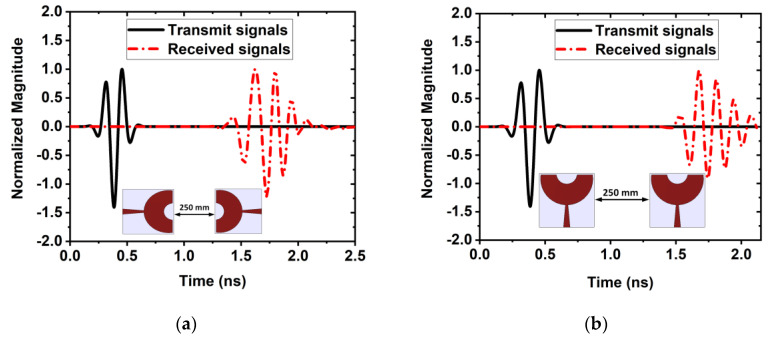
The normalized magnitude of the: (**a**) Face-to-face setup; (**b**) Side-by-side setup.

Consequently, it is decided that, with a slight alteration, the prototype can emit a small pulse. The normalized magnitude scenario of the face-to-face and side-by-side setups is illustrated in Figure 10a,b. However, it is seen that, in a face-to-face scenario, the received pulses are nearly equal to the transmitted pulses in respect to the side-by-side setup, for the highly directive radiation. So, this setup is used in the breast imaging system. Another parameter of the antenna fidelity factor (FF) is expressed by the following formula [42]:

(1) $$FF=\max\frac{\int_{-\infty }^{+\infty }T(t)R(t-\tau )dt}{\sqrt{\int_{-\infty }^{+\infty }\left|T{\left(t\right)}^{2}\right|dt\;\int_{-\infty }^{+\infty }\left|R{\left(t\right)}^{2}\right|dt}}$$

Here, *T(t)* and *R(t)* denote the transmitted and received pulses, respectively. The FF is calculated by the MATLAB programming language, and the values are 82% and 81% for face-to-face and side-by-side setups, correspondingly. These values indicate that the proposed system has minor alteration of the pulse while transmitting UWB impulse signals.

## 5. Imaging Setup and Result Discussion

**Figure 11:**
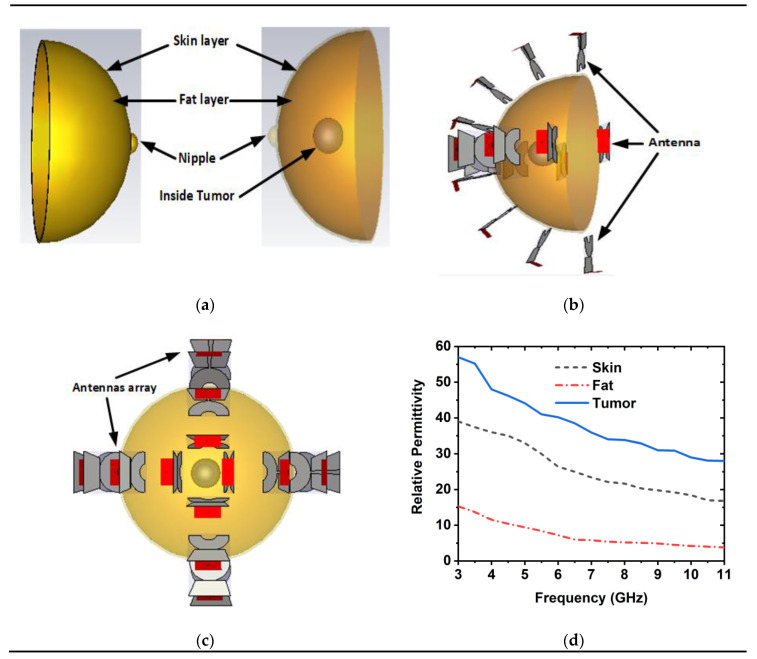
Proposed breast imaging setup: (**a**) Breast phantom tissues layer with and without tumor; (**b**) Right side view of 16-antenna array imaging setup; (**c**) Top view of 16-antenna array imaging setup; (**d**) The relative permittivity of different tissue layers of the phantom.

In this section, the imaging setup and imaging results are investigated. The performance is investigated based on different parameters together with analyzing the backscattering signal, far-field directivity, near field directivity (NFD), and S-parameters. The proposed imaging setup is presented in Figure 11a,c. To investigate the 16-antenna array performance, a breast phantom is used for identifying breast tumors. A 16-antenna array consists of 16 proposed antennas, which are placed horizontally and vertically around the breast phantom. In the 16-antenna array imaging scenario, an eight-antenna array is positioned horizontally, and the other eight-antenna array is positioned vertically, neighboring the breast phantom with the same distance from each other. In this scanning procedure, a single antenna acts as a transmitter, which is transmitting microwave pulses, and the remaining 15 antennas act as receivers, which are receiving the backscattered signal. The procedure is repetitive for every 16 antennas acting as a transmitter.

**Figure 12:**
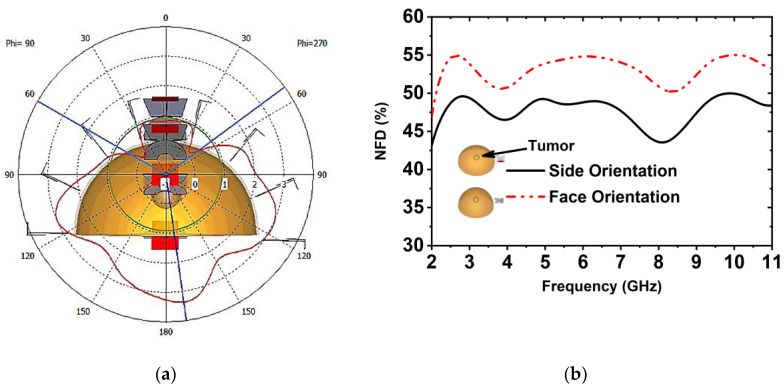
(**a**) Far-field directivity; (**b**) Near field directivity of the imaging setup.

The heterogeneous phantom has three layers: the skin layer, the fat layer, and the tumor. The phantom properties are selected based on the normal human breast tissue obtained from reduction surgeries [43]. Reference values are obtained from the literature [43,44]. However, the considered relative permittivity, conductivity, and width of the skin layer are 39, 1.5 S/m, and 2.50 mm, respectively. The radius of the breast phantom is 45 mm. The fat layer has the relative permittivity and conductivity of 15 and 0.14 S/m, respectively. In this breast scanning, the tumor tissue radius is 2.50 mm with a relative permittivity of 57 and 5 S/m of conductivity, and it has been placed at 25 mm depth inside the phantom. The dielectric properties of the breast tissues with tumor are presented in Table 3. The stated properties of different tissue layers are in a single frequency of 3.00 GHz. The skin, fat, and tumor relative permittivity versus frequency is presented in Figure 11d. The total of 16 × 15 = 240 scanned location has been assessed for a whole scanning. The microwave signals are propagated through the breast phantom. Figure 12a demonstrated the far-field directivity of the antenna. It is observed that the radiation is propagating, covering the whole phantom.

**Figure 13:**
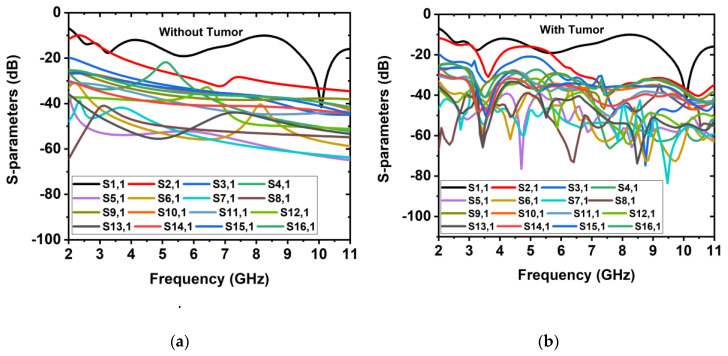
S-parameters of the arrays setup: (**a**) Without the presence of tumor; (**b**) With the presence of tumor inside breast phantom.

The NFD is the ratio of emitted power by the transmitting antenna, and the emitted power received over the surface, which can be expressed by the following formula [45]:

(2) $$NFD=\frac{P_{f}}{P_{T}}$$

where *P_f_* denotes the power emitted inside the phantom, and *P_T_* denotes the emitted power over the surface of the phantom. Figure 12b demonstrates the NFD of the antenna with the proposed imaging setup with a tumor. It is found that NFD intended for the offered imaging setup is approximately 56% when antenna setup is as face orientation. Besides, it also observed that NFD is about 49% when antenna setup is as side orientation. This means that for the proposed imaging setup, about 56% power is emitted through the breast tissue. Figure 13 represents the S-parameters (S_1,1_ to S_16,1_) response of the healthy breast (i.e., without tumor) and unhealthy breast (i.e., with a tumor) when antenna one is excited and the remaining 15 antennas are receiving the scattered signals. S-parameters means scattering parameters of the antenna. The digit associated with S represents the antenna number (i.e., 1 means antenna no. 1, 2 means antenna no. 2, 3 means antenna no. 3, … 16 means antenna no. 16, and so on). However, S_2,1_ represents backscattering signals from antenna 1; received by antenna 2, S_3,1_ represents backscattering signals from antenna 1; received by antenna 3, S_12,1_ represents backscattering signals from antenna 1; received by antenna 12, and so on.

**Figure 14:**
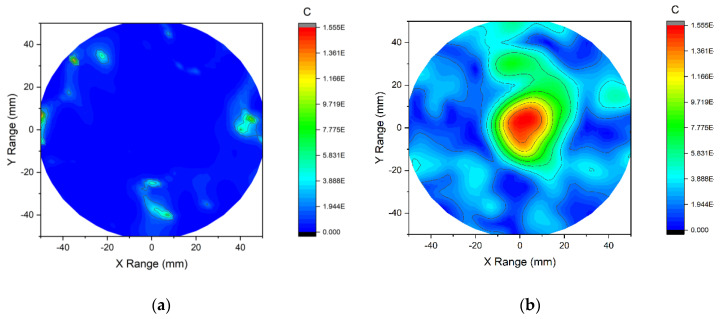
Reconstructed Images of the breast phantom: (**a**) Phantom without tumor; (**b**) Phantom with tumor.

The reflection response of the transmitter antenna is comparatively unchanged in the imaging setup. There is a significant distortion of the backscattering signal of the two graphs in Figure 13a,b. Due to the absence of the tumor, the highest peak resonance frequency is recorded about −62 dB. In contrast, in the presence of the tumor, the peak resonance is approximately −82 dB in the operating frequency band. The scattered signals are different due to the higher dielectric properties of the tumor compared to the normal breast tissues. Thus, this indicates that the antenna array system setup with the proposed antenna can be a suitable candidate for breast imaging to identify the tumor by investigating backscattering signals proficiently. Then, the backscattered signal data are collected from the imaging setup and analyzed, processed by using the IC-DAS image reconstruction algorithm [1] using the MATLAB platform. After processing the data by the algorithm, the target imaging result is illustrated in Figure 14. The reconstructed images of the breast phantom without tumor are presented in Figure 14a and the phantom with the tumor is presented in Figure 14b. It is examined that the target tumor has been visibly detected with a red color. However, it is noticeable that our proposed system can be a decent candidate for microwave breast imaging to identify the tumor by examining the backscattering signals proficiently. A comparative study of the stated antennas with the proposed antenna is listed in Table 4. However, the comparison parameters are antenna type, dimension, operating frequency range, fractional bandwidth (FBW), gain, scanning position, application, etc. Lastly, the proposed antenna is better than other reported antennas in terms of wider bandwidth, higher FBW, and stable gain with respect to dimension. The proposed system has a 16-antenna elements array, which covers 16 × 15 scanned points that help to get substantial imaging data points and produces high-resolution imaging compared to other systems reported in the literature.

## 6. Conclusions

In this article, a compact planar UWB antenna is designed, fabricated, and inspected for breast tumor imaging to detect tumors in the breast. The antenna is compact in dimension: 0.30λ × 0.31λ × 0.011λ, where λ is the wavelength of the lowest operating frequency, and it has attained an operating bandwidth of 8.70 GHz (2.30–11.00 GHz, 130.82% fractional bandwidth) with a maximum of 5.80 dBi gain, as well as stable radiation characteristics. The simulated and experimental outcomes are evaluated to validate the performance of a single antenna with UWB features. The antenna demonstrates outstanding performance in frequency-domain and time-domain performance for the face-to-face setup with a higher fidelity factor and shows good near-field directivity (NFD). A breast imaging platform with 16 antennas is designed. A breast phantom with a tumor is investigated, analyzed, and processed by using the IC-DAS algorithm for evaluating the imaging performance, and tumor is detected. Finally, it is observed that the proposed antenna is a good candidate for initial breast tumor identification through microwave imaging.

## Figures and Tables

**Table 1:** All optimized parameters of the proposed prototype.

Parameters	Size (mm)	Parameters	Size (mm)
*S_w_*	42	*l_1_*	17.72
*S_L_*	41	*l_2_*	30
*d_1_*	40	*l_3_*	18.38
*d_2_*	15	*l_4_*	42
*L_f_*	17	*h*	17
*W_f_*	5		

**Table 2:** Different structure designs comparison table.

Different Structure Designs	Operating Band (GHz)	Bandwidth (GHz)	Gain (dBi)
Normal patch design	2.26–3.00	0.74	2.50
Slotted patch design	2.30–7.00	4.70	4.50
Proposed design	2.26–11.00	8.74	5.48

**Table 3:** The dielectric properties of the human breast tissues with tumor at 3.00 GHz frequency.

Name of the Tissues	Relative Permittivity ( $\varepsilon_{r}$)	Conductivity ( σ)
Skin	39	1.5
Fat	15	0.14
Tumor	57	5

**Table 4:** Performance comparison outcomes among the reported antennas and the proposed antenna.

Ref. No	Antenna Type	Dimension ^1^(λ^3^)	Operating Frequency (GHz)	FBW (%)	Gain (dBi)	Elements /Positions	Frequency/Time Domain	Phantom and Tumor Object	Applications
[17]	Planar inverted cone antenna	0.26 × 0.27 × 0.002	1.00–10.00	163.63	8.00	Not available	Frequency domain	Not available	Ultrawide-band
[18]	Combinat-ions of Monopole antenna	0.78 × 0.78 × 0.005	3.10–10.60	109.48	10.00	Not available	Time domain	Not available	Ultrawide-band
[19]	Volcano smoke antenna	Not available	2.00–15.00	152.94	Not reported	Not available	Frequency domain	Not available	Ultrawide-band
[1]	Side slotted Vivaldi antenna	0.47 × 0.39 × 0.015	2.80–7.00	85.71	6.50	9 element antennas array, 8 × 50 scanned position	Frequency and Time domain	Heteroge-nous phantom and 2 tumors object	Microwave breast imaging
[4]	CPW feed EBG structure antenna	0.78 × 0.45 × 0.016	3.10–7.60	84.11	9.50	2 element antennas array, 2 × 120 scanned position	Frequency domain	Commercial phantom and one tumor object	Microwave breast imaging
[13]	Rectangular slotted patch antenna	0.25 × 0.27 × 0.018	3.49–12.00	109.87	5.76	7 element antennas array scanning	Frequency domain	Simulated phantom and one tumor object	Microwave breast imaging
[14]	Antipodal Vivaldi antenna	0.40 × 0.40 × 0.016	2.50–8.00	104.76	7.20	9 element antennas array, 50 × 8 scanned position	Frequency domain	Laboratory based phantom and 2 tumor objects	Microwave breast imaging
[16]	Slotted planar patch antenna	0.51 × 0.61 × 0.018	3.50–15.00	124.32	5.50	4 × 4 single element	Frequency domain	Simulated phantom and one tumor object	Microwave breast imaging
[27]	Slotted antipodal Vivaldi antenna	0.33 × 0.33 × 0.013	3.01–11.00	125.92	7.20	2 element antennas array, 2 × 50 scanned position	Time domain	Simulated phantom, one tumor object	Microwave breast imaging
[30]	antipodal Vivaldi antenna	0.33 × 0.33 × 0.013	2.50–11.00	125.92	7.20	16 element antennas array, 16 × 15 scanned position	Frequency domain and time domain	Simulated phantom, one tumor object	Microwave breast imaging
[32]	Side slotted Vivaldi antenna	0.45 × 0.38 × 0.008	1.54–7.00	127.86	8.50	2 element antennas array scanning	Frequency domain	Commercial phantom and one tumor object	Microwave breast imaging
[34]	CPW feed monopole antenna	0.53 × 0.5 × 0.01	2.00–4.00	66.67	5.20	16 elements array, 16 × 15 scanned position	Time domain	Simulated phantom, one tumor object	Microwave breast imaging
[40]	Tapered and transmiss-ion loaded antenna	0.33 × 0.27 × 0.016	2.00–8.00	120.00	4.89	16 element antennas array, 16 × 15 scanned position	Time domain	Lab based phantom and single tumor object	Microwave breast imaging
**Pro-** **posed**	Semi-circle shaped planar antenna	0.30 × 0.31 × 0.011	2.30–11.00	130.82	5.80	16 element antennas array, 16 × 15 scanned position	Frequency and time domain	Simulated phantom, one tumor object	Microwave breast imaging

^1^ λ is the wavelength of the lowest operating frequency.
